# Air Pollution Detection and Sports Club Environmental Responsibility Based on the RBF Neural Network

**DOI:** 10.1155/2022/3693310

**Published:** 2022-08-24

**Authors:** Zijian Guo, Huijun Lin, Zebo Qiao

**Affiliations:** ^1^Guangdong University of Finance & Economics, Sports Dept, Guangzhou 510320, Guangdong, China; ^2^Competitive Sports Academy, Guangdong Sports Vocational and Technical College, Guangzhou 510500, Guangdong, China

## Abstract

With the rapid development of soft computing technology, various models and comprehensive analysis methods are emerging one after another, and new theories and research results continue to emerge, showing great strength and development potential in actual theoretical research and engineering applications. This paper analyzes the air pollution detection and environmental responsibility of sports clubs based on RBF neural networks, constructs the corresponding neural network algorithm, and simulates and analyzes the data. In the process of simulation design, we adjust the weight and threshold of the network according to the error performance of the network to realize the functions required by the system. Different models were used to predict the concentration of air pollutants in typical cities. At the same time, a meta-analysis method was used to conduct a preliminary discussion on the impact of air pollutants on the health of the Chinese population, and some research results were obtained. In the past years, Chinese sports clubs have also built a solid social environmental protection system around the related environmental protection responsibilities of sports clubs. The research on green environmental monitoring has improved people's awareness of environmental responsibility and provided technical support for the green development of sports clubs.

## 1. Introduction

Combining the characteristics and advantages of soft computing, this topic introduces the research background, significance, and current research status of soft computing in China [1]. According to the soft computing methods and algorithms used in this paper, the principles, models, structures, and properties of fuzzy systems and neural networks are described, as well as the philosophical and methodological basis of the integration of soft computing methods, and the integration mode of the above methods is analyzed [2]. At the same time, this article examines the impact of urban transportation resource positioning on the development of green cities, analyzes the multifactor and high-dimensional independent variables in the path positioning of rail transit systems, and also highlights the development direction of positioning research based on soft computing methods [3]. Then, based on the analysis of the above attribute model, a multilayer model method based on the genetic algorithm and the neural network is proposed, which completes the selection of factor variables in the urban rail transit trunk line location model [4]. Finally, according to actual application requirements, the application problems are studied, and the location of urban rail transit lines is analyzed in detail [5]. We explore the spatial and temporal distribution characteristics of China's air pollutants, three prediction models, carry out air pollution detection in representative cities in a large area, and compare and analyze the monitoring effects and accuracy to find a more ideal monitoring model, and we study the effects of air pollution on the health of residents in China [6]. At the same time, based on the three prediction models of the BP neural network, the vector machine, and the wavelet support vector machine, six types of pollutants in representative cities in typical regions of China were modeled and analyzed [7]. Taking China's existing sports club models and operating conditions as research objects, we adopt literature research methods, combining quantitative research and comparative analysis and analyze the development status of Chinese sports clubs through descriptive statistics [8]. Using the principles of systemicity and practicability, the efficiency index system of professional football clubs is constructed, and the importance of each index is analyzed [9]. At the same time, the environmental protection responsibility of sports clubs is helpful to the implementation of this research, and the corresponding theoretical basis is used to solve the problem of today's sports clubs performing environmental protection responsibilities [10]. Only by paying attention to and assuming the social responsibility related to economic benefits, and constantly raising new issues and discussing them, can sports clubs embody their greatest social value [11].

## 2. Materials and Methods

### 2.1. Design of Soft Computing Algorithm

In the learning process, the weights and thresholds of the network are adjusted according to the error performance of the network to achieve the required functions. The basic iterative formula of the steepest gradient algorithm based on the gradient method is expressed as follows:

(1) $$\begin{array}{c} \text{x}\left(\text{k}+1\right)=\text{x}\left(\text{k}\right)+\mathrm{\alpha g}\left(\text{k}\right). \end{array}$$

Among them, the network output error can generally adopt the method of the mean square error, the mean absolute error, and the sum of the mean square error.

The input layer is composed of some perception units, which connect the network with the external environment. The second layer is the only hidden layer in the network. Its function is to perform a nonlinear transformation from the input space to the hidden layer space. According to the nature of the Gaussian function, it is not difficult to find that when the input signal is close to the center of the basis function, the hidden layer node will produce a larger output so that this type of network has the ability of local approximation.

The basis functions used are as follows:

(2) $$\begin{array}{c} \text{f}\left(\text{x}\right)=\text{exp}{\left(\frac{-\text{x}}{\sigma }\right)}^{2},\\ \text{f}\left(\text{x}\right)=\frac{1}{{\left(\sigma^{2}+{\text{x}}^{2}\right)}^{\alpha }},\alpha >0,\\ \text{f}\left(\text{x}\right)={\left(\sigma^{2}+{\text{x}}^{2}\right)}^{2},0<\beta <1. \end{array}$$

The above functions are all radially symmetric, but the most commonly used is the Gaussian function (Gaussian function), as shown in the following formula:

(3) $$\begin{array}{c} {\text{R}}_{\text{i}}\left(\text{x}\right)=\text{exp}\left[-\frac{\left\|x-c_{i}\right\|}{2\sigma_{i}^{2}}\right]. \end{array}$$

In formula (3), ||*x*−*c*_*i*_|| is the norm of the vector *x*−*c*_*i*_, which usually represents the distance between *x* and *c*_*i*_, *R*_*i*_ (*x*) has a unique maximum value at *c*_*i*_, as ||*x*, and with the increase of −*c*_*i*_||, *R*_i_ (*x*) quickly decays to zero. For a given input *x* ∈ *R*^*n*^, only a part of the input near the center is activated.

The principle of radius selection is to make the receptive fields of all RBF units cover the entire training sample. For each cluster center ci, the corresponding radius σ_*i*_ is determined by equation (4), which means that the radius σ_i_ is equal to the average distance between *c*_*i*_ and training samples belonging to the class, as shown in the following formula:

(4) $$\begin{array}{c} \sigma_{\text{i}}=\frac{1}{{\text{M}}_{\text{i}}}\underset{\text{x}\in \Gamma_{\text{i}}}{\sum }\sqrt{{\left(\text{x}-{\text{c}}_{\text{i}}\right)}^{\text{T}}\left(\text{x}-{\text{c}}_{\text{i}}\right)}. \end{array}$$

Another way to select σ_*i*_ is to find the average distance between the centers of the N nearest neighbors of each cluster center *c*_*i*_ as the value of σ_*i*_, as shown in the following formula:

(5) $$\begin{array}{c} \sigma_{\text{i}}=\frac{1}{\text{N}}\overset{\text{N}}{\underset{\text{j}=1}{\sum }}\left\|{\text{c}}_{\text{i}}-{\text{c}}_{\text{j}}\right\|. \end{array}$$

### 2.2. Research on the Status Quo of the Performance of Environmental Protection Responsibilities of Sports Clubs

China's air quality monitoring cities are all over the Chinese mainland, but the distribution is extremely uneven. There are relatively few monitoring cities and monitoring points in a certain area of China, while many monitoring cities and monitoring points are relatively dense in some areas. This is consistent with the distribution of Chinese towns and population in the northwest and the southeast. The reason for this phenomenon is the mild climate in the southeast region, and it was the first region in China to vigorously develop its economy. However, the northwestern region is arid and rainless, and the large-scale distribution of the Gobi and deserts makes it difficult for people to survive, so it is relatively backward. This article has compiled a questionnaire on the environmental responsibility of Chinese club operations. The list of the questionnaire validity test is shown in Table 1.

The reliability of the questionnaire was retested. A total of 16 people were selected from 33 people, and the same questionnaire was sent to them after 10 days; if *r* = 0.84 determined by the two surveys of the questionnaire, its reliability is acceptable. The validity is evaluated on the basis of expert judgment, which proves the feasibility and rationality of the structure of the questionnaire.

## 3. Results

### 3.1. Temporal Characteristics of Air Pollutants in Green Cities

**Figure 1:**
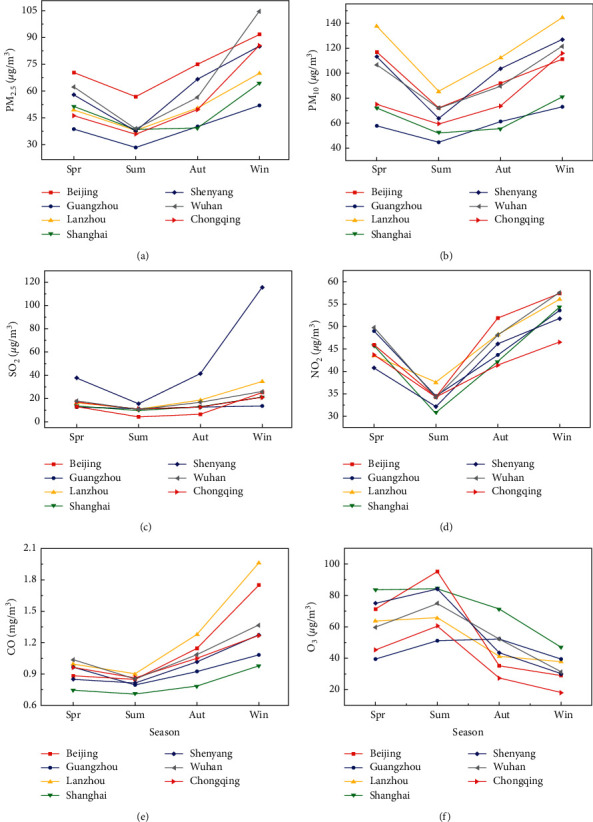
2017–2020 China's representative city air pollution seasonal changes; (a) PM_2.5_ change; (b) PM10 change; (c) SO_2_ change; (d) NO_3_ change; (e) CO change; (f) O_3_ law of change.

By calculating the monthly average concentration of air pollutants in major cities in China from 2017 to 2020, the annual change pattern of major cities in China is determined. The seasonal changes in air pollution in major cities in China from 2017 to 2020: PM_2.5_, PM_10_, NO_2_, and SO_2_ show the similar change pattern is that seasonal changes in winter and spring are significantly higher than those in summer and autumn. The annual changes of PM_2.5_, SO_2_, and CO show a “U”-shaped curve, reaching a peak in January, 39.78 *μ*g/m^3^ and 1.57 mg/m^3^, respectively. January is the month with the lowest average temperature of the year in China, but the winter heating period in northern cities is still dominated by coal, and incomplete combustion of coal will emit a large amount of CO. O_3_ presents an inverted U-shaped curve, with serious pollution from April to September and peaks in May. In most areas of China, the sunshine duration is longer from April to September, which is conducive to the formation of O_3_. PM_10_ presents a typical bimodal curve, reaching peaks in March and December at 109.13 *μ*g/m^3^ and 116.16 *μ*g/m^3^, respectively. March is the most frequent period of dusty weather in China, and December is the winter heating period, which shows that the pollution of coarse particles is caused by natural dust and man-made coal. NO_2_ presents an atypical bimodal curve, with peaks in March and December at 48.41 *μ*g/m^3^ and 39.29 *μ*g/m^3^, which are related to the warming of coal burning in China in winter and are also affected by the effects of climate change on pollutants. The impact of regional transport caused by diffusion and spring winds is hazardous. In order to further reduce emissions, the competent authority should continue to vigorously promote winter coal-to-gas heating and promote clean energy vehicles. To solve the problem of PM2.5, the traffic flow must be restricted. At present, effective methods are adopted to limit travel, restrict vehicle purchases, and vigorously develop new energy vehicles. In addition, complete public transportation facilities can well solve the problem of citizens' travel under the condition of vehicle restrictions, which should be paid attention to. Regarding the pollution of other chemical substances in the atmosphere, we should continue to strengthen the control of industrial dust rates. Firstly, clean production should be advocated, and corresponding policies should be introduced to restrict industries and factories that generate a lot of dust; secondly, measures such as water mist adsorption and installation of filter membranes should be adopted to suppress the rate of industrial dust, thereby reducing particulate pollutants in the atmosphere and reducing air pollution. In addition to SO_2_ pollution, in addition to daily cleaning work, it is necessary to strengthen the remediation of exposed garbage. We should strengthen the mechanization of road dust areas, do a good job in dust control and environmental protection, and consolidate and improve the cleaning quality of road sections; in road cleaning operations, we should increase the number of various types of auxiliary machinery and reduce the workload of cleaning personnel. Through calculation, the seasonal change rule of the city is obtained, as shown in Figure 1.

Figure 1 shows that the seasonal variation of PM_2.5_ in winter and autumn is the lowest among the representative cities, while the seasonal variation of PM_2.5_ in other cities is highest in winter and lowest in summer. The seasonal variation of PM10 in different cities is different, which means that the annual variation of PM10 in the city is basically a “U”-shaped curve, and the pollution level is relatively high in January and November-December. Except for a certain province, the annual changes in SO_2_ are relatively mild. The small peak in January and December is closely related to the implementation of China's desulfurization and emission reduction policies. The peak in March may be related to unfavorable weather conditions at that time.

### 3.2. Spatial Distribution Characteristics of Air Pollutants in Green Cities

**Figure 2:**
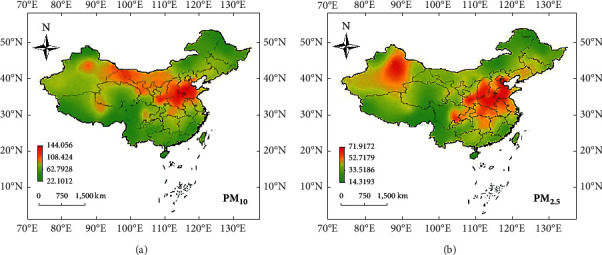
The spatial distribution characteristics of PM_10_ and PM_2.5_ in China from 2017 to 2020: (a) the spatial distribution characteristics of PM_10_; (b) the spatial stepwise characteristics of PM_2.5_.

The spatial distribution of air pollutants in a certain area is mainly monsoon climate, and the amount of precipitation is lower than that of southern cities. A certain area has the characteristics of strong cold air and sandy dust weather. The highest concentration of PM_10_ is 155.05 *μ*g/m^3^, and the highest concentration of PM_2.5_ is 85.09 *μ*g/m^3^. Among the 364 cities monitored, the compliance status of China's PM_10_ and PM_2.5_ primary and secondary standard boundaries is shown in Figure 2.

It can be seen from Figure 2 that the spatial distribution of PM_10_ and PM_2.5_ in China has obvious characteristics of first-class quality areas. The above-mentioned spatial distribution characteristics are mainly related to the monsoon climate of a certain area. The precipitation is lower than that of southern cities, and a certain area is affected by strong cold air, which brings sand and dust weather, making the area a high pollution center of coarse particles. Moreover, the large amount of industrial emissions makes the pollution of coarse and fine particles more serious. The distribution of PM_2.5_ pollutants is mainly related to major industrial emissions, and climate and geographical conditions are not conducive to its spread.

### 3.3. Analysis of the Status Quo of the Performance of Environmental Protection Responsibilities of Sports Clubs

**Figure 3:**
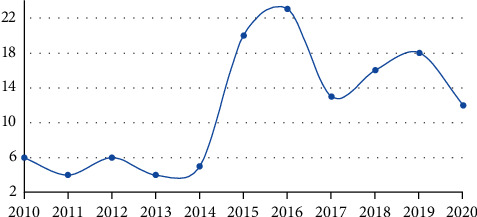
Environmental protection responsibilities of running clubs in China in the past ten years.

The number of environmental responsibility reports of major sports clubs in China is gradually increasing, showing a steady trend year by year. Judging from the number of environmental protection responsibilities disclosed by running clubs in China over the years, reports on the environmental responsibilities of running clubs have increased year by year and have gradually stabilized. Environmental protection responsibilities of running clubs in China are shown in Figure 3.

It can be concluded that running clubs are aware of the importance and necessity of fulfilling their environmental responsibilities, and the disclosure of their environmental responsibilities by relevant media and news reports has become more and more common, which has increased their enthusiasm for assuming environmental responsibilities.

In China's 34 regions, running clubs in only 31 regions have fulfilled their environmental protection responsibilities, and most regions have only fulfilled them once. This shows that running clubs can have a broader geographical scope and a broader development of environmental protection responsibilities and establish their own in China. Statistics of places where running clubs fulfill their environmental responsibilities are shown in Table 2.

Table 2 shows that if clubs want to continue to operate in the future, internationalization is an indispensable part of them so that they can have global influence like the NBA. Running clubs can start from a certain province and gradually distribute the impact on the entire society.

It can be said that in the past ten years, in order to fulfill its environmental responsibility, the club has served society in various ways. Statistics of the specific performance times of running clubs in fulfilling their environmental responsibilities are shown in Table 3.

It can be seen from Table 3 that in recent years, the club has fulfilled its environmental protection responsibilities in many aspects such as complying with the law and improving the quality of its own infrastructure. Other aspects are reflected in charity activities, community contributions, and cultural exchanges.

According to the specific possibilities of operating clubs to fulfill their environmental responsibilities, as shown in Table 4, they are divided into independent and cooperative groups (clubs, players, and sponsors).

In addition to the club itself, players and coaches also recognize that they are in the running club. As a member of the club, they have an obligation to contribute to society. Therefore, we can see that the most important way to fulfill the responsibilities of clubs to environment is cooperation between clubs, supplemented by independent development.

## 4. Discussion

### 4.1. Environmental Protection Management of Sports Club Events

The development of outdoor sports in China is in full swing, and environmental problems exposed have become increasingly prominent, directly affecting the reputation and development of sports competitions. As a result, the environmental management of the competition has been put on the agenda. The stakeholders of the competition organization are actively looking for different solutions, such as balancing the competition experience with environmental protection goals and trying to transform the content of environmental management into highlights, and hence, environmental management has become more and more important [12]. Driven by China's economic development and strategic requirements of healthy China, environmental protection has become an important research topic for China to achieve national health [13]. At present, the development of the nation has entered a stage of diversified development and mature development. Environmental protection is the most worrying issue among people's needs for healthy life. Among them, the negative environmental problems caused by road races have become the most important environmental protection theme. The government must strengthen the environmental responsibility of the host organization, implement environmental management regulations, and raise appropriate entry barriers [14].

How the organizer faces environmental problems that may arise during the event plays an important role in the sustainable development of the event [15]. It is also the most effective resource and opportunity for the organizer to maximize the benefits. The long-term development of the event depends on the organizer's influence on the event. Only by organizing the competition in an environmentally friendly manner, the road race can show the trend of rapid and healthy development in the future, which is not only worthy of self-interest. Cross-country races are also one of the most well-known events in the Chinese running circle, and the competition activities are mainly held in the countryside. Compared with urban runners, the feeling of walking in natural environment is a different state. Therefore, the destruction of the natural environment and the waste of disposable items in the event will attract more attention.

**Figure 4:**
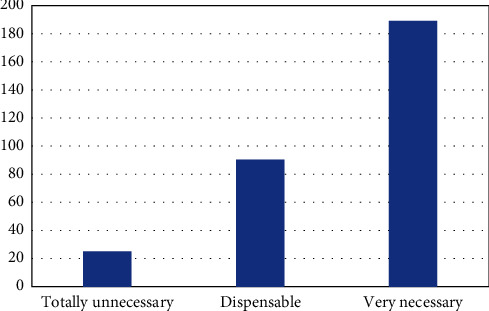
The need for environmental promotion of sports events.

Through the outdoor road race, the participants accepted the challenge, achieved health, participated in social activities, and received pleasant feeling. They fully recognize the requirements of personal physical and mental health and hope to minimize the impact on the external environment in this process, effectively achieve the balance between personal health and external environmental health, and truly achieve the deep integration of fitness and health [16]. Participants' direct feedback on the environmental protection promotion of the event is shown in Figure 4.

It shows that 55% of people think that outdoor sports companies and event organizers should be responsible for environmental protection, and 63% of people think that organizers' publicity activities are more useful, which shows that the organizers should take the important responsibility for the environmental management of the event.

### 4.2. Analysis of the Status Quo of Environmental Protection Management of Foreign Sports Events

The development of foreign sports events and the formation of sports habits are relatively early, and the corresponding experience in environmental management of sports events is also relatively rich and mature. This article analyzes the status quo of environmental management of sports events from the perspective of stakeholders.

The United States nongovernmental organization (CRS) began to supervise the environmental protection of marathons and major sports in the early years. It is an organization specifically designed for the environmental protection of sports events. It is the world's leading sustainable sports certification program, providing three services: assistance in management, certification, and rewards. CRS standard version 4.2 is currently available, as shown in Table 5.

The purpose of its establishment is to encourage and promote all sectors of society to be responsible for society and environment in the process of various sports.

The organizers themselves have a mature understanding of the importance of environmental protection. We also learned from the official website of CRS that all large-scale outdoor running activities in specific areas actively participate in this certification and continue to promote environmental protection measures in the organization and implementation of activities according to this standard. After the certification list is announced, the marathon will be held in specific areas. It has been certified for five competitions, of which, the most recent two times were green level, 1 time was silver level, and 2 times were qualified. The Fliggy Marathon has won golden certification for 3 sessions.

### 4.3. Analysis of the Problems in the Environmental Management of Sports Club Events

In recent years, with the rapid development of the sports industry and consumption, China's national fitness and health concept has been deeply rooted in the hearts of the people. Various outdoor games are flourishing. Environmental management issues are also emerging endlessly, and participants in all aspects of the event have more or less responsibilities. At present, the most important core issue in the environmental management of outdoor street activities in China is the lack of government legislation and the lack of top-down binding management benchmarks, resulting in “unguided” management. All stakeholders are consciously looking for solutions, but they have no scale, no system, and no limitations.

The first and most well-known environmental problem of outdoor road races is the transportation of solid waste, and international marathons are often criticized as garbage dumps. The hosting of sports events mainly exerts a pressure effect on the urban environment and has the characteristics of insecurity, dynamic change, and superposition. In order to promote the sustainable development of the urban ecological environment, this article proposes some measures to strengthen the evaluation of the ecological sustainability of the host city before the competition, strengthen the monitoring of the ecological environment of the host city during the competition, and strengthen the restoration of the ecological environment of the host city after the competition.

### 4.4. Strategies to Enhance the Environmental Responsibility of Sports Clubs

Relevant government agencies formulate laws and regulations to regulate and restrict the environmental behavior of operators and stakeholders. Two-level mandatory projects and advocacy projects are established. The classification, division, and classification methods of different events are flexibly introduced to realize the balanced development of management and incentive. According to the degree of difficulty, it can be divided into the leisure time, standard level, challenge level, and examination level. According to different organizers, it can be divided into activities organized by the government, social groups, enterprises, and individuals. According to different regions, it can be divided into business, adventurous, and restrictive activities. In the organization and management of activities, we must recognize the importance of the coordinated development of the ecological environment and outdoor road transport activities. The organization plan includes content related to environmental protection and strengthens the audit to verify the identity of participants through the intelligent event information system to reduce hidden risks. We should formulate effective technical means and obtain good competitive experience and environmental protection measures at the same time.

Under the dual management of government coordination and event organizers, competitors' participation concepts and environmental awareness will continue to improve. In addition to putting forward self-discipline requirements for their own environmental behaviors, they should also play the role of personal supervision, supervise the activities of the activity organization and other participants, and put forward positive complaints and feedback on problems. As a source of interest for the event organization, participants must recognize the necessity of environmental protection for the reputation and future development of the event from the perspective of sustainable development. This is the need for long-term benefits of an event.

## 5. Conclusion

This article discusses how to use soft computing to solve the problem of location selection of urban road traffic resources. This paper expounds on the basic theory of the gray system and God, analyzes various influencing factors, and conducts simulation and experimental research according to actual application requirements. Based on data coverage analysis, this article tests the operating efficiency of sports clubs. In the field of empirical research, sports clubs are the research objects. In different times, sports clubs have different connotations and meanings. Therefore, the environmental protection responsibility of sports clubs is researched, and relevant theoretical foundations are used to solve the problems existing in the performance of environmental protection responsibilities of sports clubs today.

## Figures and Tables

**Table 1:** List of the questionnaire validity test (*N* = 33).

	Very effective	Efficient	Invalid
Questionnaire content	20	13	0
Questionnaire structure	23	10	0

**Table 2:** Statistics of places where running clubs fulfill their environmental responsibilities.

Province	Frequency	Province	Frequency	Province	Frequency
Region 1	32	Region 13	1	Region 25	1
Region 2	16	Region 14	2	Region 26	1
Region 3	17	Region 15	0	Region 27	1
Region 4	9	Region 16	2	Region 28	1
Region 5	6	Region 17	2	Region 29	1
Region 6	7	Region 18	2	Region 30	1
Region 7	4	Region 19	0	Region 31	1
Region 8	4	Region 20	2	Region 32	1
Region 9	2	Region 21	0	Region 33	1
Region 10	3	Region 22	2	Region 34	1
Region 11	1	Region 23	0	Unknown	14
Region 12	3	Region 24	1	—	—

**Table 3:** Statistics of the specific performance times of running clubs in fulfilling their environmental responsibilities in the past ten years.

Years	Infrastructure	Charity events	Audience contributions	Community contributions	Cultural dissemination	Educational activities	Environmental protection activities
2010	—	7	—	—	—	—	—
2011	—	2	—	—	—	—	2
2012	—	6	—	2	—	—	—
2013	—	3	—	—	—	—	—
2014	—	4	—	0	2	—	—
2015	2	8	7	4	1	2	—
2016	1	4	3	2	10	0	—
2017	—	4	—	3	6	2	—
2018	—	7	—	0	4	1	0
2019	2	5	—	2	3	6	—
2020	—	1	2	4	3	1	2
Total	5	51	12	19	29	12	4
Proportion	3.79%	38.64%	9.09%	14.39%	21.97%	9.09%	3.03%

**Table 4:** In the past ten years, running clubs have performed their social responsibilities.

Years	Independent development	Collective development
2010	2	6
2011	0	2
2012	3	5
2013	1	3
2014	3	4
2015	5	13
2016	3	21
2017	9	4
2018	10	6
2019	3	13
2020	7	7
Total	46	84

**Table 5:** 2008–2020 CRS green certification competition list.

Serial number	Tournament list
1	2018 Bank of America Marathon
2	2018 City Open
3	2018 NCAA Women's Big Four Basketball Championship
4	2017 NCAA Men's Four Basketball Championship
6	2017 City Open
6	Bank of America Marathon 2016
7	2016 IAAF International Athletics Championships
8	2016 Ironman Triathlon Competition
9	2016 10 km Coastal Running Race
10	2016 U.S. Olympic Track and Field Trials
11	2010 Half Marathon of an energy company
12	A university running event in 2009
13	2008 triathlon competition in a state

## Data Availability

The data used to support the findings of this study are available from the corresponding author upon request.
